# Adult subventricular zone neural stem cells as a potential source of dopaminergic replacement neurons

**DOI:** 10.3389/fnins.2014.00016

**Published:** 2014-02-10

**Authors:** John W. Cave, Meng Wang, Harriet Baker

**Affiliations:** ^1^Brain and Mind Research Institute, Weill Cornell Medical CollegeNew York, NY, USA; ^2^Burke Medical Research InstituteWhite Plains, NY, USA

**Keywords:** subventricular zone, olfactory bulb, neural stem cells, adult neurogenesis, dopaminergic, cell replacement therapy

## Abstract

Clinical trials engrafting human fetal ventral mesencephalic tissue have demonstrated, in principle, that cell replacement therapy provides substantial long-lasting improvement of motor impairments generated by Parkinson's Disease (PD). The use of fetal tissue is not practical for widespread clinical implementation of this therapy, but stem cells are a promising alternative source for obtaining replacement cells. The ideal stem cell source has yet to be established and, in this review, we discuss the potential of neural stem cells in the adult subventricular zone (SVZ) as an autologous source of replacement cells. We identify three key challenges for further developing this potential source of replacement cells: (1) improving survival of transplanted cells, (2) suppressing glial progenitor proliferation and survival, and (3) developing methods to efficiently produce dopaminergic neurons. Subventricular neural stem cells naturally produce a dopaminergic interneuron phenotype that has an apparent lack of vulnerability to PD-mediated degeneration. We also discuss whether olfactory bulb dopaminergic neurons derived from adult SVZ neural stem cells are a suitable source for cell replacement strategies.

## Introduction

Parkinson's Disease (PD) is one of the most prevalent neurodegenerative disorders and afflicts ~1% of the population over 60 years of age in industrialized nations (Nussbaum and Ellis, 2003). PD affects several regions of the nervous system that result in many clinical symptoms (Braak et al., 2004; Poewe, 2008). The most characteristic symptoms, however, are motor system impairments that include tremor, rigidity, and bradykinesia. The pathology underlying these motor symptoms is a progressive degeneration of dopaminergic neurons in the substantia nigra pars compacta. These dopamine neurons modulate control of motor function through axonal projections to the putamen and caudate divisions of the striatum. Standard clinical care is focused on the functional preservation of this nigrostriatal circuit. Pharmacological approaches with L-DOPA and dopamine receptor agonists can ameliorate most motor symptoms, particularly in the early stages after a clinical diagnosis. As the disease progresses, however, medication regimens become less effective and surgical strategies, such as deep brain stimulation, can provide symptomatic relief. Both the pharmacological and surgical interventions ultimately fail though because they do not stop the progressive degeneration of midbrain dopaminergic neurons.

Cell replacement strategies are a fundamentally different type of therapy because they introduce new dopaminergic neurons that can restore striatal dopamine levels. Clinical trials engrafting human fetal ventral mesencephalic tissue containing dopaminergic neuronal progenitors into the striatum demonstrated, in principle, that this approach can provide substantial long-lasting improvement of motor symptoms (Widner et al., 1992; Defer et al., 1996; Freed et al., 2001; Mendez et al., 2002; Olanow et al., 2003). Some patients in these trials improved post-operatively to a point where L-DOPA therapy was no longer required, but others experienced negligible long-term improvement and, in some cases, severe graft-induced dyskinesias developed and required additional surgical intervention.

## Ideal features of replacement cell sources

Analyses of the clinical trials identified several features of replacement cells that are essential for favorable functional outcomes (Wijeyekoon and Barker, 2009; Lindvall and Bjorklund, 2011). These features include establishing replacement cell sources that are autologous, or at the least allogenic, since immunosuppression appears to improve engrafted cell survival and clinical outcomes (Olanow et al., 2003). The survival rate of transplanted cells is an important practical issue and sources that provide replacement cells with higher survival rates reduce the total amount of donor material required. Significant clinical benefit is estimated to require the survival of at least 100,000 engrafted dopaminergic neurons per putamen (Hagell and Brundin, 2001). Clinical improvement is also more likely achieved with sources that can be used as dissociated cell suspensions. Unlike solid tissue grafts, cell suspensions can readily disperse and evenly innervate target areas. Studies of engraftments in both humans and rodents indicate the uneven and patchy reinnervation of the putamen promotes the development of adverse dyskinesia side effects (Ma et al., 2002; Carlsson et al., 2006; Maries et al., 2006). The risk of dyskinesias is also reduced with replacement cell sources that can be prepared by standardized protocols that minimize the presence of undesired phenotypes. The presence of serotonergic progenitors in fetal mesencephalic tissue, for example, is believed to contribute to the incidence of transplant-related dyskinesias (Politis et al., 2012). Consistent with this possibility, serotonergic neurons transplanted into the striatum of rodent PD models exacerbate dyskinesias induced by L-DOPA (Carlsson et al., 2007).

## Embryonic and induced pluripotent stem cell sources

The ideal source of replacement cells for PD has not been established (reviewed in Meyer et al., 2010). Fetal mesencephalic tissue is not practical because of the difficulty in standardizing donor tissue and ethical concerns of how to generate the necessary amounts of material required for widespread clinical use. Stem cells, however, are a promising source of replacement cells that meet the requirements for creating favorable clinical outcomes (discussed above). Several studies have demonstrated the feasibility of stem cell based therapies using embryonic stem cells (Bjorklund et al., 2002; Kim et al., 2002; Takagi et al., 2005; Roy et al., 2006; Rodriguez-Gomez et al., 2007; Cho et al., 2008; Sanchez-Pernaute et al., 2008; Friling et al., 2009). These studies used a variety of differentiation protocols, but they all showed that transplants of embryonic stem cell-derived dopaminergic neurons into either rodent or primate PD model systems both survive and integrate into the host circuitry to provide significant functional recovery. Embryonic stem cells face several important challenges, however, before they can be considered for human clinical trials. These challenges include developing higher efficiency protocols for dopaminergic neuron production, improving survival of engrafted cells, and methods to completely remove contaminating cell types responsible for tumor or teratoma formation observed in animal trials.

Induced pluripotent stem cells are another potential source of replacement cells. Their broad differentiation potential resembles embryonic stem cells, but the ability to derive these cells from adult tissue avoids the moral and ethical issues of using embryonic stem cell lines. Furthermore, induced pluripotent stem cells are a potential autologous source of replacement cells since they can be generated from a patient's own cells. Recent reports indicate that dopaminergic neurons derived from induced pluripotent stem cells can survive and provide functional improvement in rodent PD model systems (Wernig et al., 2008; Hargus et al., 2010; Swistowski et al., 2010; Rhee et al., 2011; Chang et al., 2012; Sundberg et al., 2013). Advancing induced pluripotent stem cells to a clinical setting faces many of same challenges confronting the use of embyronic stem cells, including improvement in the dopaminergic neuron yields and prevention of tumor formation in the recipient.

## Replacement cells derived from the adult SVZ

### Adult neural stem cells

Neural stem cells are an alternative source of replacement neurons. These cells lack the pluripotent potential of embryonic and induced pluripotent stem cells, but they also do not have the same teratoma or tumor forming potential. Fetal tissue is a rich source of neural stem cells that can generate a wide array of neuronal phenotypes, but as discussed above, the use of fetal tissue is fraught with moral and ethical problems. By contrast, adult neural stem cells do not have these issues, although they typically generate a more limited number of neuronal phenotypes. In the adult mammalian brain, there are two well-established neurogenic niches: the hippocampal subgranular zone and the subventricular zone (SVZ) of the lateral ventricles (Gage et al., 2008; Seki et al., 2011). Neurons generated in both of these regions are able to integrate into pre-existing neural circuitry, which makes them attractive candidates for cell replacement therapy. Progenitors generated in the subgranular zone migrate to the dentate gyrus and become excitatory glutamatergic granule cells. Subgranular zone neural stem cells are located within the interior of the hippocampus, however, and extracting them without damaging the hippocampus is difficult. SVZ neural stem cells almost exclusively produce inhibitory GABAergic inhibitory interneuron progenitors that migrate to the olfactory bulb glomerular and granule cells layers (production of a small number of excitatory neurons has also been reported in Brill et al., 2009). SVZ neural stem cells reside within the walls of the lateral ventricle and, by penetrating non-eloquent parts of the brain in humans, they can be endoscopically harvested, expanded *in vitro* and differentiated into both neuronal and glial progenitors (Westerlund et al., 2005). Thus, adult SVZ neural stem cells are a potential clinically feasible source of autologous replacement neurons.

### Transplantation of adult SVZ cells into the striatum

Several studies have homochronously transplanted either SVZ tissue explants or suspensions of either neurospheres or dissociated cells into the adult rodent striatum (Table 1) (Lois and Alvarez-Buylla, 1994; Herrera et al., 1999; Zhang et al., 2003; Meissner et al., 2005; Richardson et al., 2005; Seidenfaden et al., 2006; Chen et al., 2007a,b; Shim et al., 2007; Deleidi et al., 2011). Similar to other sources of donor cells, the survival rates of transplanted SVZ cells were consistently low. Also similar to clinical trials with fetal tissue, migration of transplanted SVZ cells away from the injection site was greater with dispersed SVZ cultures or neurospheres than with engraftments of tissue explants.

**Table 1:** **Summary of studies transplanting adult SVZ-derived cells into the adult striatum**.

**Study**	**Type of SVZ tissue transplanted**	**Donor species and genotype**	**Modifications to donor SVZ tissue prior to transplantation**	**Recipient species and phenotype**	**Analysis of recipient animals post-transplantation**	**Key findings**
Lois and Alvarez-Buylla, 1994	~100 μm diameter explants	NSE-LacZ mice	None	Wild-type mice	30 days	No migration of surviving cells outside of transplantation site
Herrera et al., 1999	~150 μm diameter explants	NSE-LacZ mice	None	Mice with kainic acid lesions	2, 4, 6, and 8 weeks	Limited migration of transplanted cells Vast majority of surviving NSE-LacZ expressing cells were astrocytes
Zhang et al., 2003	Dissociated cell suspensions	Wild-type rat	Neural progenitor cells cultured for 8 days in media with FGF2, horse and calf serum; cells were labeled with either BrdU or DiO	Wild-type rats	7 and 28 days	Substantial migration away from transplantation site Majority of BrdU and DiO labeled cells expressed either MAP2 or NeuN, few expressed GFAP
Meissner et al., 2005	Neurosphere suspensions	βActin-CMV-GFP mice	Neurospheres were cultured for 5 passages in the presence of EGF and FGF2	Mice with unilateral 6-hydroxydopamine OHDA lesions	1 month	Low survival rate of transplanted cells ~7% and ~2% of surviving cells expressed TH Improved motor performance to apomorphine and amphetamine challenges
Richardson et al., 2005	Dissociated cell suspensions	Wild-type rat	Neural progenitor cells isolated by a Percoll gradient, cultured for 1 week in media containing FGF2, then 6 days in media containing retinoic acid without FGF2; cells were labeled with BrdU	Rats with unilateral 6-hydroxydopamine OHDA lesions	2 weeks	Many surviving transplanted cells maintain Nestin expression, but none expressed NeuN Improved motor performance to amphetamine challenge
Seidenfaden et al., 2006	~100 μm diameter explants	βActin-CMV-GFP mice	None	Wild-type mice	3 weeks and 6 months	Limited migration and low survival rate of transplanted cells Most surviving cells expressed glial marker genes
Chen et al., 2007b	Neurosphere suspensions	Wild-type rat	Neural progenitor cells isolated by a Percoll gradient protocol, cultured for 2 weeks in media containing EGF and FGF2; cells were labeled with BrdU	Wild-type rat	6 weeks	Substantial migration away from transplantation site Most surviving cells expressed GFAP and none expressed NeuN
Chen et al., 2007a	Neurosphere suspensions	Wild-type rat	Neural progenitor cells isolated by a Percoll gradient protocol, cultured for 2 weeks in media containing EGF and FGF2; cells were labeled with BrdU	Rats transduced with AAV_1/2_ vectors to over-express either BDNF or FGF2 in the striatum	6 and 12 weeks	AAV_1/2_-FGF2 improved survival of transplanted cells Most surviving transplanted cells expressed glial marker genes AAV_1/2_-BDNF increased percentage of surviving cells expressing NeuN
Shim et al., 2007	Dissociated cell suspensions	Wild-type rat	Neurospheres were cultured in media containing EGF, FGF2, and PDGF for over 2 months; retroviral transduction to over-express Nurr1 and Mash1	Rats with unilateral 6-hydroxydopamine OHDA lesions	2, 4, and 6 weeks	Substantial number of surviving transplanted cells expressed MAP2, TH and VMAT2 Improved motor performance to amphetamine challenge
Deleidi et al., 2011	Dissociated cell suspensions	rTA/Oct4 mice	Neurospheres were expanded for 14 days before being converted to induced pluripotent stem cells and then differentiated via 5 stage protocol; SSEA1+ cells were removed by cell sorting prior to transplantation	Rats with unilateral 6-hydroxydopamine OHDA lesions	4, 6, and 8 weeks	Substantial number of surviving transplanted cells express TH Improved motor performance to apomorphine and amphetamine challenges

An unexpected and consistent finding in the rodent studies with SVZ-derived cells was the meager differentiation and survival of transplanted neurons. Using either cell suspensions or tissue explants as donor material, many of the surviving cells displayed glial phenotypes and very few expressed markers of mature neurons. The poor neuron yields were surprising since the adult SVZ normally produces large numbers of neuronal progenitors (Luskin, 1993; Lois and Alvarez-Buylla, 1994). Analyses of SVZ progenitor cultures expanded *in vitro* found a large percentage of cells expressed glial marker genes prior to transplantation (Richardson et al., 2005; Chen et al., 2007b), suggesting that *in vitro* culture conditions may preferentially select for glial precursors. An exception is a study by Zhang and co-workers that reported achieving a high percentage (~70%) of neuronal precursors in SVZ cultures prior to transplantation (Zhang et al., 2003). A majority of cells that survived a month after transplantation were found to have a mature a neuronal phenotype and only a few cells expressed the glial marker GFAP. The reasons for higher levels of neuronal generation and survival in this study are unclear. The interactions between adult SVZ neural stem cells and their surrounding niche are complex and only just becoming understood (reviewed in Ihrie and Alvarez-Buylla, 2011). Despite their poor neuronal differentiation and survival, transplants of SVZ-derived cells into the host striatum did provide some functional recovery in 6-hydroxydopamine lesioned rodent PD models a month after surgery (Meissner et al., 2005; Richardson et al., 2005). The extent of the improvement was variable, but it does suggest that introduction of glia is not deleterious and may produce some short-term symptomatic improvement.

### Dopaminergic differentiation of SVZ progenitors

In addition to the poor differentiation and survival of neurons with SVZ-cell transplants, few, if any, of the detectable neurons displayed dopaminergic features. The human clinical trials clearly showed that integration and survival of dopaminergic neurons is essential for sustained long-term functional improvement. Embryonic and induced pluripotent stem cells can be directed toward a dopaminergic cell fate by culturing them with appropriate growth factors and morphogens (Kriks and Studer, 2009; Boyer et al., 2012). Similar protocols can significantly improve neuronal progenitor yields in cultured SVZ cells, but they do not direct these cells to a dopaminergic cell fate (Deleidi et al., 2011). These protocols can generate dopaminergic neurons, however, if the SVZ progenitors are first converted into pluripotent stem cells by *Oct4* over-expression (Deleidi et al., 2011). *Oct4* has a central role in stem cell pluripotency and differentiation, and is sufficient to reprogram adult neural stem cells into pluripotent stem cells (Pesce and Scholer, 2001; Kim et al., 2009). About 5% of the total cells in culture can be directed to adopt a midbrain dopaminergic neuronal phenotype when SVZ progenitors are reprogrammed by *Oct4* over-expression. Transplantation of cultures with these reprogrammed dopaminergic neurons into 6-hydroxydopamine lesioned rats produced significant and reproducible functional improvement for up to 2 months after the procedure (Deleidi et al., 2011).

An alternative genetic engineering strategy for improving dopaminergic neuron yields is to over-express transcription factors that specify cell fate during normal development. This approach has been successfully used with both embryonic and induced pluripotent stem cells (Chung et al., 2002, 2005; Kim et al., 2002; Andersson et al., 2006; Martinat et al., 2006; Friling et al., 2009; Sanchez-Danes et al., 2012; Theka et al., 2013). Both *Mash-1* and *Nurr1* genes encode transcription factors that are involved in the specification of midbrain dopaminergic neurons (Park et al., 2006). Retroviral-mediated over-expression of these transcription factors can direct a substantial percentage (~37%) of the total number of cultured adult SVZ cells to express dopaminergic neuronal markers (Shim et al., 2007). Transplantation of these cells in 6-hydroxydopamine lesioned rats also produces robust functional improvement.

### SVZ/olfactory bulb dopaminergic phenotype as a potential replacement cell source

Adult SVZ neural stem cells normally generate dopaminergic interneurons and both *Mash1* and *Nurr1*-family transcription factors are important for specification of these interneurons (Cave and Baker, 2009). Although the over-expression of *Mash1* and *Nurr1* was intended to generate dopaminergic neurons with a midbrain phenotype, this strategy may have generated either A16 olfactory bulb dopaminergic neurons or hybrid A16 neurons. The ability of the dopaminergic neurons generated by Shim and co-workers to significantly improve functional performance in the rodent PD model, raises the possibility that either an A16 or a hybrid A16 dopaminergic neuron is a viable alternative for cell replacement strategies.

Non-midbrain dopaminergic neurons are not typically considered for replacement cells in PD. This is due, in part, to studies of transplanted dopaminergic neurons from non-mesencepahlic brain regions. Grafts of diencephalic dopaminergic neurons from either rodent or human embryonic hypothalamic tissue poorly integrated into the host striatum and provided no significant functional benefit in PD models (Abrous et al., 1988; Zuddas et al., 1991; Frodl et al., 1994). Furthermore, previous clinical trials grafting tissue containing catecholaminergic neurons from non-brain regions, including the adrenal medulla, carotid body, retinal epithelium and sympathetic ganglion neurons, demonstrated either only marginal or no significant benefit. In addition, morbidities associated some procedures, such as autografts of either adrenal medulla and sympathetic ganglia, have further discouraged exploration of non-midbrain phenotypes as replacement cells (reviewed in Wijeyekoon and Barker, 2009).

Olfactory bulb dopaminergic neurons have several favorable attributes, however, that are distinct from these previously tested non-midbrain phenotypes. Like other adult-generated neurons, olfactory bulb dopaminergic progenitors can integrate into pre-existing neural circuitry. Mature neurons are likely able to innervate large areas of striatum since they have processes that typically extend for over 200 μm and, in some cases, over 700 μm (Kiyokage et al., 2010). Like their counterparts in the substantia nigra, a prominent electrophysiological property of olfactory bulb dopaminergic neurons is a capacity to generate auto-rhythmic action potentials (Pignatelli et al., 2005; Guzman et al., 2009). Unlike the substantia nigra, however, olfactory bulb dopaminergic neurons are not vulnerable in PD-mediated neurodegeneration (Huisman et al., 2004). Consistent with this reduced vulnerability, olfactory bulb dopaminergic neurons lack α-synuclein deposits in rodents over-expressing this protein (Ubeda-Banon et al., 2010). Several PD patients surviving at least 10 years after intrastriatal grafts of fetal mesencephalic tissue have shown accumulation of α-synuclein in grafted neurons (Kordower et al., 2008a,b; Li et al., 2008, 2010; Mendez et al., 2008). Whether this accumulation adversely affects the function or survival of replacement neurons is not known, but it does suggest that replacement neurons of the midbrain phenotype are susceptible to PD in the long-term. Replacement neurons with the olfactory bulb dopaminergic phenotype, however, are expected to be less vulnerable to this pathology.

### Challenges for developing adult SVZ cells as replacement cells

Developing adult SVZ cells into a clinically suitable autologous source of replacement neurons faces several challenges. One challenge is to enhance survival rates of engrafted cells. Studies with embryonic and induced pluripotent stem cells, for which long-term survival is also an issue, have indicated that treating the donor cells with antioxidants or growth factors can increase survival rates (Nakao et al., 1994; Apostolides et al., 1998; Espejo et al., 2000; Hoglinger et al., 2001; Andereggen et al., 2009). The presence of neurotransmitters in the culture conditions may improve the ability to expand and differentiate cells with neuronal phenotypes since neurotransmitters can modulate proliferation, migration and survival of SVZ-derived cells in the endogenous SVZ neurogenic niche (reviewed in Young et al., 2011). In addition, pre-conditioning recipients by administering growth factors may also be an important strategy to improve long-term survival (Chen et al., 2007a). Elucidating culture conditions that promote neuronal progenitor expansion and survival will be critical for developing standardized preparations to generate clinically suitable replacement cells from adult SVZ neural stem cells.

A second challenge is the suppression of glial progenitor proliferation and survival. SVZ neural stem cells prodigiously generate neuronal progenitors in their endogenous niche, but most of the progenitors generated by these cells either do not to survive or they adopt alternative fates when cultured *in vitro* and transplanted into the striatum. Furthermore, proportional levels of glial production increase with either the age of the SVZ neural stem cell donor or the length of time that the stem cells are cultured (Gritti et al., 2009). Since most PD patients are advanced in age, the propensity of autologously derived SVZ stem cells to produce glia is an important challenge to address. Protocols for *in vitro* expansion of SVZ progenitors typically use growth factor combinations that include epidermal growth factor (EGF) (Deleyrolle and Reynolds, 2009). The inclusion of this growth factor, however, may be detrimental for neuronal progenitor production since stimulation of EGF receptors promotes glial progenitor proliferation and differentiation in the SVZ (Aguirre and Gallo, 2007; Gonzalez-Perez and Quinones-Hinojosa, 2010; Gonzalez-Perez and Alvarez-Buylla, 2011). Alternatively, culture media may require other growth factors to suppress glial differentiation. Expansion of neurospheres derived from the adult SVZ in media containing both EGF and FGF9 enhances neuronal proliferation while diminishing both oligodendrocyte and astrocyte progenitor production (Lum et al., 2009). Supplementing *in vitro* culture conditions with Noggin may also enhance suppression of glial phenotypes. SVZ neural stem cells and neural progenitors express BMP receptors and antagonism of these receptors by Noggin inhibits glial differentiation (Lim et al., 2000). Furthermore, cell-sorting techniques can facilitate the removal of glial progenitors from SVZ-derived cultures prior to transplantation. Recent studies with human pluripotent progenitors showed that glial progenitors can be successfully isolated from mixed cultures based on co-expression of CD44 and CD184 (Yuan et al., 2011).

A third challenge is developing efficient methods to produce dopaminergic neurons. Treatment either with growth factors, morphogens, signaling molecules, or chromatin modifying agents does not direct SVZ-derived progenitors toward a dopaminergic fate. By contrast, significant dopaminergic neuron production has been only achieved through genetic modification of SVZ-derived progenitors (Shim et al., 2007; Deleidi et al., 2011). These genetic engineering methods, however, come with some caveats. Reprogramming of SVZ neural stem cells into pluripotent cells prior to dopaminergic differentiation introduces the same safety concerns associated with induced pluripotent stem cell strategies. Retroviral transduction of SVZ progenitors to over-express transcription factor genes, such as *Nurr1* and *Mash1*, avoids the concerns of genetic reprogramming, but retroviruses are questionable for clinical application since they can increase the risk of tumor formation by disrupting endogenous gene expression upon their insertion into the host genome (Yi et al., 2011). Genetically engineering SVZ progenitors for cell replacement therapy in PD will likely require one of the other available methods to transduce cells (Lowry and Plath, 2008).

Even though over-expression of *Nurr1* and *Mash1* has provided the best yield of SVZ-derived dopaminergic neurons to date, only a subset of transduced cells adopted a dopaminergic phenotype. This partial induction may reflect the heterogeneity within the SVZ stem cell niche. Specific subsets of olfactory bulb interneurons are generated from the neural stem cells that are spatially localized in distinct regions of the adult SVZ (Kelsch et al., 2007; Merkle et al., 2007; Young et al., 2007; Brill et al., 2009). Further studies are required to establish whether adult SVZ neural stem cells from spatially distinct regions are limited in their capacity to generate dopaminergic progenitors *in vitro*. In addition to transcription factors, heterogeneous expression of microRNAs may also be instrumental in shaping the *in vitro* potential of adult SVZ neural stem cells to generate dopaminergic neurons (De Chevigny et al., 2012). Further studies are also required to address whether the dopaminergic neurons produced with SVZ-derived progenitors either adopt an olfactory bulb, midbrain or a hybrid dopaminergic phenotype. As discussed above, the olfactory bulb phenotype may be clinically advantageous for replacement cells, but one concern is that olfactory bulb dopaminergic interneurons also co-express of GABA (Kiyokage et al., 2010). The effect of GABA in these potential replacement cells is difficult to predict, but recent studies suggest that nigral dopaminergic neurons inhibit striatal output through the co-release of both dopamine and GABA (Tritsch et al., 2012). Consistent with this observation, about 10% of dopaminergic neurons in substantia nigra pars compacta are reported to co-express mRNA for the GABA biosynthetic enzyme glutamate decarboxylase 65 (Gonzalez-Hernandez et al., 2001). Thus, the co-expressed GABAergic phenotype in olfactory bulb dopaminergic interneurons may be more of a similarity than a difference with nigral dopaminergic neurons. Studies in rodent PD animal models are needed, however, to demonstrate whether the olfactory bulb or a hybrid dopaminergic phenotype can provide functional recovery without producing deleterious side effects.

## Conclusions

The adult SVZ is a promising source of neural stem cells to generate dopaminergic neuronal progenitors for cell transplant strategies in PD. Since these stem cells can be endogenously harvested from patients, they are a potential autologous source of replacement cells. As discussed in this review, however, there are several important technical issues to be addressed in order for adult SVZ neural stem cells to be competitive source for clinically suitable dopaminergic neurons.

### Conflict of interest statement

The authors declare that the research was conducted in the absence of any commercial or financial relationships that could be construed as a potential conflict of interest.
